# Local alkylating chemotherapy applied immediately after 5-ALA guided resection of glioblastoma does not provide additional benefit

**DOI:** 10.1007/s11060-017-2649-8

**Published:** 2017-11-14

**Authors:** William Sage, Mathew Guilfoyle, Catriona Luney, Adam Young, Rohitashwa Sinha, Donatella Sgubin, Joseph H. McAbee, Ruichong Ma, Sarah Jefferies, Rajesh Jena, Fiona Harris, Kieren Allinson, Tomasz Matys, Wendi Qian, Thomas Santarius, Stephen Price, Colin Watts

**Affiliations:** 1 Division of Neurosurgery, Addenbrookes Hospital, Cambridge University NHS Foundation Trust, Cambridge, UK; 2 Division of Neurosurgery, Azienda Ospedaliera Nazionale SS, Antonio e Biagio e Cesare Arrigo, Alessandria, Italy; 3 Department of Clinical Neurosciences, John van Geest Centre for Brain Repair, University of Cambridge, Cambridge, UK; 4 Department of Neurosurgery, John Radcliffe Hospital NHS Foundation Trust, Oxford, UK; 5 Department of Oncology, Addenbrookes Hospital, Cambridge University NHS Foundation Trust, Cambridge, UK; 6 Department of Histopathology, Addenbrookes Hospital, Cambridge University NHS Foundation Trust, Cambridge, UK; 7 Department of Radiology, Addenbrookes Hospital, Cambridge University NHS Foundation Trust, Cambridge, UK; 8 Cambridge Cancer Trial Centre, Cambridge Clinical Trials Unit – Cancer Theme, Cambridge University NHS Foundation Trust, Cambridge, UK; 9 Division of Neurosurgery, Department of Clinical Neurosciences, Addenbrookes Hospital, University of Cambridge, Hills Road, Box 167, Cambridge, CB2 0QQ UK

**Keywords:** Neurosurgery, Glioma, 5-Aminolevulinic acid, Carmustine

## Abstract

Grade IV glioma is the most common and aggressive primary brain tumour. Gross total resection with 5-aminolevulinic acid (5-ALA) guided surgery combined with local chemotherapy (carmustine wafers) is an attractive treatment strategy in these patients. No previous studies have examined the benefit carmustine wafers in a treatment programme of 5-ALA guided resection followed by a temozolomide-based chemoradiotherapy protocol. The objective of this study was to examine the benefit of carmustine wafers on survival in patients undergoing 5-ALA guided resection. A retrospective cohort study of 260 patients who underwent 5-ALA resection of confirmed WHO 2007 Grade IV glioma between July 2009 and December 2014. Survival curves were calculated using the Kaplan–Meier method from surgery. The log-rank test was used to compare survival curves between groups. Cox regression was performed to identify variables predicting survival. A propensity score matched analysis was used to compare survival between patients who did and did not receive carmustine wafers while controlling for baseline characteristics. Propensity matched analysis showed no significant survival benefit of insertion of carmustine wafers over 5-ALA resection alone (HR 0.97 [0.68–1.26], p = 0.836). There was a trend to higher incidence of wound infection in those who received carmustine wafers (15.4 vs. 7.1%, p = 0.064). The Cox regression analysis showed that intraoperative residual fluorescent tumour and residual enhancing tumour on post-operative MRI were significantly predictive of reduced survival. Carmustine wafers have no added benefit following 5-ALA guided resection. Residual fluorescence and residual enhancing disease following resection have a negative impact on survival.

## Introduction

WHO Grade IV glioma is the most common and aggressive primary brain tumour and presents amongst the most formidable challenges of any cancer. Each year in the UK ~ 4300 new cases of brain or central nervous system (CNS) cancers are diagnosed, around 7 per 100,000 population. Although brain tumours account for < 2% of all primary tumours they are responsible for 7% of the years of life lost from cancer before age 70 (ONS 2006 Series MB1 No. 34). If the burden of disease is considered in terms of the average years of life lost per patient, brain tumours are one of the most lethal cancers with over 20 years of life lost [1].

Surgery remains a mainstay of treatment and is essential in establishing a diagnosis. As surgical techniques have improved, the importance of obtaining a gross total resection is increasingly recognized and is being incorporated into European guidelines [2, 3]. Prospective cohort data suggest that radical resection, defined by the absence of post-operative contrast enhancing disease, improves survival (over 9 months) [4], that gross total resection (GTR) but not incomplete resection of glioblastoma prolongs survival (6 months) in the era of radiochemotherapy [5] and also that multiple resections for patients with glioblastoma can prolong survival [6].

Complete resection of contrast enhancing disease is only achieved in a minority of patients because identifying the margin between tumour and adjacent brain intraoperatively is frequently challenging. 5-aminolevulinic acid (5-ALA) is a heme precursor that after oral administration results in accumulation of porphyrins in epithelial and neoplastic cells, including malignant glioma. Under ultraviolet light from an operating microscope tumour tissue is distinctly fluorescent compared with surrounding white matter and provides the surgeon with real-time intraoperative guidance during resection [7]. A phase III randomised controlled trial has shown that 5-ALA guided resection of malignant glioma is associated with a significant increase in the proportion of patients where complete resection of enhancing tumour is achieved and improved progression-free survival at 6 months [8].

Local chemotherapy with biodegradable carmustine impregnated wafers inserted into the surgical resection cavity is an attractive strategy to directly deliver an active agent into the brain interstitium and circumvent the limited bioavailability and the adverse effects associated with systemic administration [9]. Although two randomised trials pointed towards a treatment effect of carmustine wafers for newly diagnosed grade IV glioma [10, 11], these were shortly followed by clear evidence of significantly improved survival with combined radiotherapy and systemic temozolomide. In this context, together with improved surgical resection strategies, the efficacy of carmustine wafers remains unproven.

The objective of this study was to examine the additional benefit of carmustine wafers on survival in patients undergoing 5-ALA guided tumour resection and factors correlated with survival.

## Methods

### Data collection

Since July 2009 all patients who receive 5-ALA fluorescence guided resection and/or carmustine wafers (Gliadel) implants have been prospectively recorded at our unit. From this database we selected all patients who underwent primary resection of a radiologically suspected malignant glioma and in whom the histopathological analysis subsequently confirmed a WHO 2007 grade IV glioma, operated on between July 2009 and December 2014.

Electronic health records were accessed to retrieve additional data regarding the surgical procedure, pathological findings, and adjuvant chemo- and radiotherapy the patient received. Our unit policy is to obtain post-operative contrast enhanced cerebral magnetic resonance imaging (MRI) within 72 h from surgery. The neuroradiologists’ reports were reviewed for each patient in the cohort to determine whether any contrast enhancing tumour remained following surgery.

The location of each tumour was determined following review of relevant neuroradiology reports, operative notes, and preoperative stealth MRIs. In the event that a tumour extended into multiple regions (e.g. parieto-occipital), it was counted in the total for both involved regions. Tumour size was defined by measuring the maximum axial dimension on preoperative scans. Measurements were corroborated with neuroradiology reports, whenever possible, and placed into RTOG size groups.

The decision of intention to insert carmustine wafers was made pre-operatively and then confirmed once the intraoperative frozen section indicated a high-grade glioma and there were no contraindications such as a wide opening of the lateral ventricle. Between four and eight wafers were inserted.

Post-operative oncology treatment, if received, was classified as either palliative: radiotherapy (typically 30 Gy in six fractions), or radical radiotherapy (60 Gy in 30 fractions), or radical chemo-radiotherapy i.e. radiotherapy combined with concomitant and/or adjuvant temozolomide as per the Stupp protocol [4].

Current status of patients and dates of death were ascertained through the NHS national tracing service on 01 January 2017 i.e. a minimum of 2 years after diagnosis. The project was registered and approved with the local audit department prior to data collection (local audit number PRN 4507).

### *IDH1* and *IDH2* mutation analysis and *MGMT* methylation analysis

Immunohistochemistry for the protein product of mutant *IDH1* R132H was performed on deparaffinised sections after heat-induced antigen retrieval. In appropriate cases that did not have the common mutation, tumour samples were submitted for next-generation sequencing to look for rarer *IDH1* mutations on exon 4, codon 132, and mutations of *IDH2* on exon 4, codon 172. To determine MGMT promoter methylation status, tumour DNA was extracted and bisulphite-converted. The MGMT promoter methylation was determined by pyrosequencing of four CpG sites (CpGs 76–79) in exon 1 of the MGMT gene using the CE-Marked Therascreen MGMT Pyro Kit on a Pyromark Q24 System (Qiagen). A cut-off of 10% methylation for the four CpG sites is used to assign a glioma as methylated or unmethylated based on published data [12–14].

### Statistical analysis

The effect of 5-ALA guidance on extent of resection was measured as whether there was residual enhancing tumour on early post-operative MRI. Comparison was made using the standard chi-squared test between patients with or without 5-ALA guided resection.

Overall survival curves were calculated using the Kaplan–Meier method from the date of surgery until date of death from all causes. The log-rank test was used to compare survival curves between carmustine versus no carmustine. Cox proportional hazards regression was performed to investigate independent factors predicting survival.

To compare survival between patients who did and did not receive carmustine wafers while controlling for differences in their baseline characteristics a propensity score matched analysis was also conducted [15]. The propensity score was calculated with a logistic regression of age, sex, tumour size, presence of residual (5-ALA) fluorescence at the end of resection, and whether the lateral ventricle had been opened during the procedure. Control (i.e. no carmustine wafers) patients were then matched to patients who received carmustine wafers with an optimal matching procedure and caliper width of 0.1, without replacement.

The independent effect on survival of variables such as carmustine wafers and residual enhancement on post-operative MRI was further estimated using standardised inverse probability weighting to adjust the respective survival curves. The weighting was calculated as the actual probability of group assignment divided by the predicted group assignment based on a logistic regression of other pre- and post-operative variables.

All analysis was carried out in R (v. 3.3.0; http://www.r-project.org) using the *stats, survival*, and *MatchIt* packages. Statistical significance level was set at p < 0.05.

## Results

### 5-ALA and carmustine wafers

A total of 260 patients underwent primary 5-ALA guided resection of a confirmed grade IV glioma between 2009 and 2014; 78 (30%) of these also received carmustine wafer implants. The age, sex, and pathology of patients in each group were similar. However, tumour size (RTOG classification), completeness of resection based on residual fluorescence and MRI, and the oncological treatment patients received following surgery was significantly different between the groups (Table 1). The variation in post-operative oncological management reflects the real-world nature of the patient cohort consistent with a surgical study. NICE guidelines permit RT/TMZ (Stupp protocol) for patients with PS 0–1 so post-operative changes may alter subsequent treatment. There was an association between tumour size and presence of residual enhancement on post-operative MRI (RTOG I: 32.4% vs. RTOG II: 45.5%, p = 0.044). In addition, there was a trend to higher incidence of wound infection requiring revision surgery in the patients who received carmustine wafers (12/78 [15.4%] vs. 13/182 [7.1%], p = 0.064).

**Table 1:** Details of the patient cohort

	5-ALA; no wafers	5-ALA; with wafers	
N	182	78	
Age (years; median [IQR])	63 [10]	61 [14]	P = 0.07
Sex (% male)	65.4	70.5	P = 0.50
RTOG class (%)			P = 0.043
I	52.2	66.7	
II	47.8	33.3	
Location			P = 0.240
Frontal	31.3	32.1	
Temporal	43.4	33.3	
Parietal	27.5	41.0	
Occipital	11.0	14.1	
Residual disease (%)			
Fluorescence	45.2	25	P = 0.04
MRI	45.3	29.9	P = 0.10
Wound infection (%)	7.1	15.4	P = 0.06
Pathology (%)			P = 0.16
GBM	78	69	
GBMO	17	26.9	
GS	2.7	3.8	
GBMP	2.2	0	
Post-operative treatment (%)			P = 0.0009
None	20	11.5	
Palliative RT (30 Gy)	28.3	10.3	
Radical RT (60 Gy)	3.3	5.1	
Chemotherapy + RT (60 Gy)	48.3	73.1	
Repeat resection (%)	2.2	5.1	P = 0.25

*5-ALA* 5-aminolevulinic acid, *RT* radiotherapy, *RTOG* radiation therapy oncology group, *GBM* glioblastoma multiforme, *GBMO* GBM with oligodendroglial component, *GBMP* GBM with primitive neuroectodermal tumour (PNET) differentiation, *GS* gliosarcoma

**Fig. 1:**
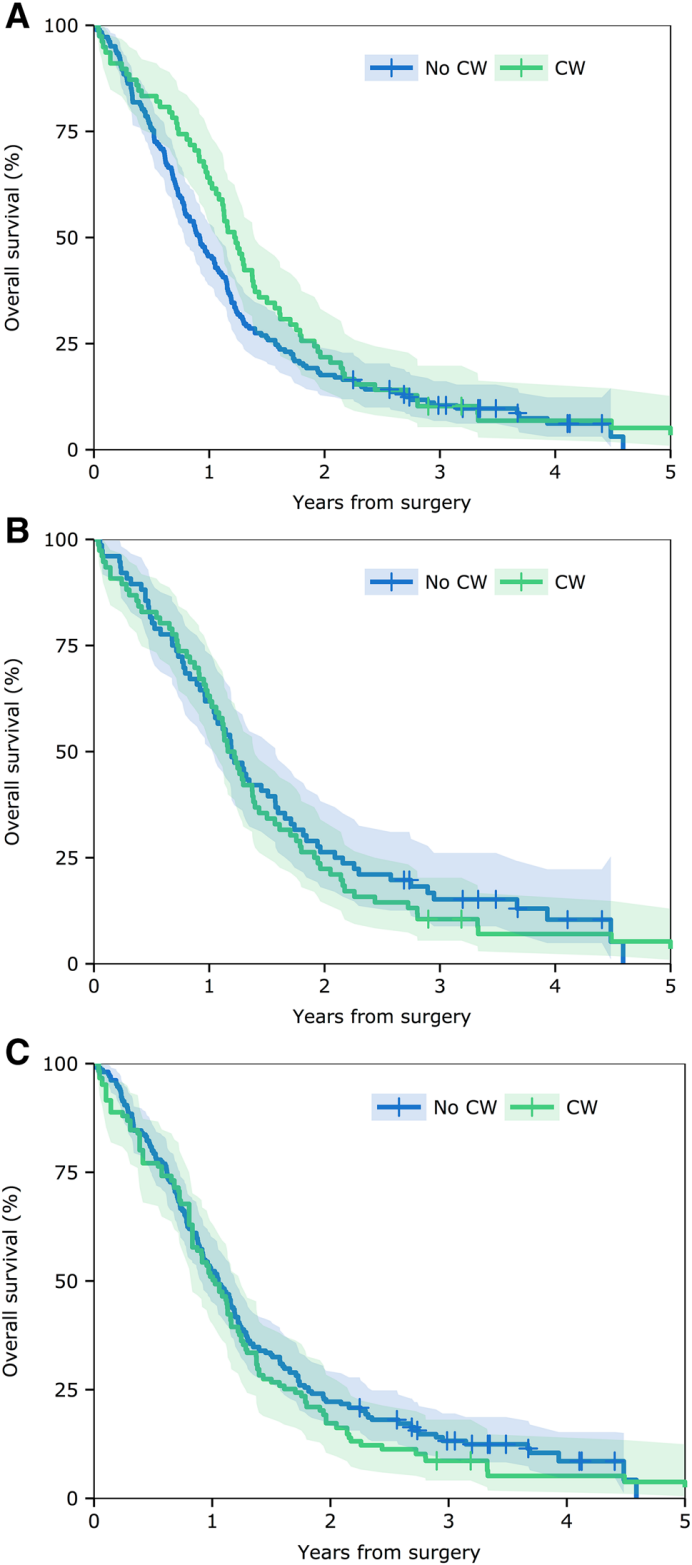
Comparison of survival for patients treated with or without carmustine wafers. Kaplan–Meier survival curves for patients having 5-ALA guided resection without carmustine wafers (No CW) and 5-ALA resection with carmustine wafers (CW). **a** Unadjusted analysis of the two patient groups. **b** Groups matched for baseline covariates by propensity score (see text for details). **c** Survival curves adjusted for baseline covariates and post-operative treatment by inverse probability weighting. *HR* hazard ratio for the log-rank test

Unadjusted comparison of survival between patients who received carmustine and those who did not, found 6 month survival was not significantly different (carmustine vs. no carmustine; 16.7 vs. 24.2%, p = 0.239). There was a significant difference at 12 months (35.9 vs. 54.4%, p = 0.009) but this did not persist at 24 months (78.2 vs. 82.4%, p = 0.338) and overall comparison of survival curves similarly found no significant difference (median 14.7 vs. 11.0 months; hazard ratio [95% CI] 0.81 [0.54–1.09], p = 0.13; Fig. 1a).

We then calculated a propensity score for the use of carmustine wafers for each patient based on pre-implantation factors comprising age, sex, tumour size, presence of residual fluorescent tumour, and whether the lateral ventricle was opened. This allowed 76 patients who received carmustine wafers to be matched to the same number of patients with similar pre-implantation features who did not receive carmustine wafers. Comparison of survival between these matched groups found no significant difference in overall survival (median 14.2 vs. 14.3 months; HR 1.10 [0.79–1.53], p = 0.59; Fig. 1b).

To examine the independent effects of both pre- and post-operative factors on survival a Cox proportional hazards regression model was constructed. Age, tumour size, residual intraoperative fluorescent tumour, residual enhancing tumour on early post-operative MRI, and adjuvant treatment were identified as significantly associated with survival, whereas use of carmustine wafers was not (Table 2).

**Table 2:** Cox-regression analysis (n = 248)

Factor	Coefficient	P value
Age (per year)	1.02	**0.009**
Sex (female vs. male)	1.03	0.86
Carmustine wafers	1.28	0.13
Residual		
Fluorescence	1.54	**0.004**
MRI	1.70	**0.0003**
Infection	1.33	0.21
Pathology (vs. GBM)		
GBMO	1.26	0.17
GS	0.90	0.80
GBMP	1.22	0.71
MGMT methylation positive (n = 128)	1.30	0.24
IDH1 (n = 128)	0.89	0.80
Post-operative treatment (vs. none)		
Palliative RT (30 Gy)	0.58	**0.01**
Radical RT (60 Gy)	0.31	**0.003**
Chemotherapy + RT (60 Gy)	0.20	< **10** ^**−10**^
Repeat resection	1.33	0.21

Bold indicate P values of < 0.05

Abbreviations as in Table 1

Correspondingly, survival curves adjusted for both pre- and post-operative factors, namely age, sex, tumour size, residual fluorescence, residual enhancing tumour on MRI, and adjuvant treatment, using standardised inverse variance weighting were essentially indistinguishable between the group of patients who received carmustine wafers and those who did not (median 12.3 vs. 12.6 months; HR 1.12 [0.85–1.50], p = 0.44; Fig. 1c).

### Residual tumour

**Fig. 2:**
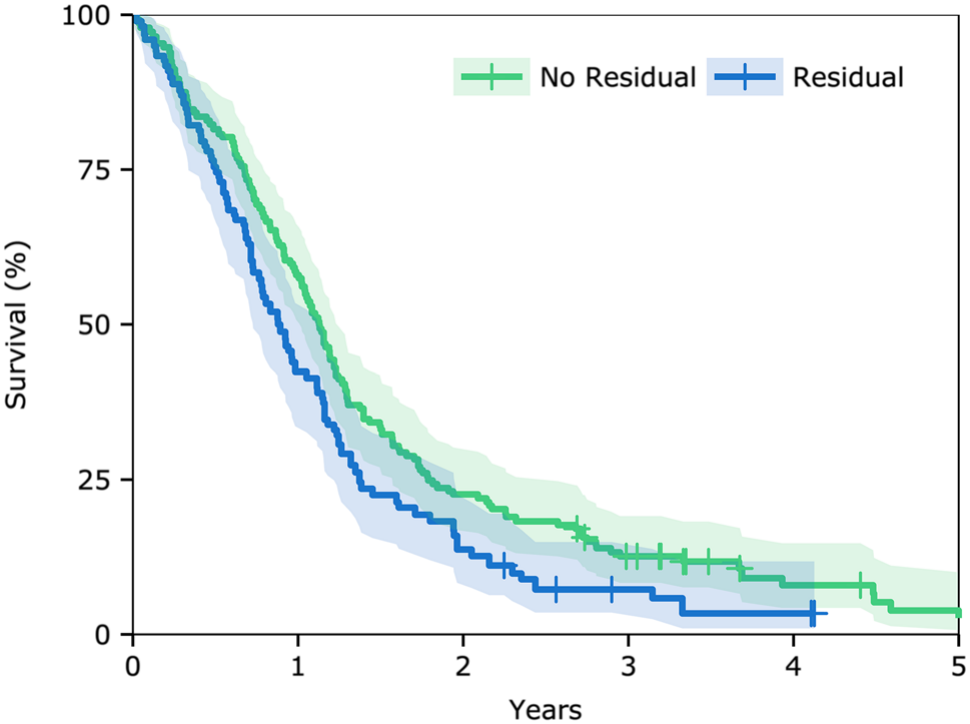
Effect on Survival of Residual Enhancing Tumour on MRI. Kaplan–Meier survival curves, adjusted for baseline covariates and post-operative treatment, for patients with and without evidence of residual enhancing tumour on post-operative MRI within 72 h from surgery. *HR* hazard ratio for the log-rank test

The Cox regression analysis showed that both intraoperative residual fluorescent tumour and residual enhancing tumour on post-operative MRI were significantly predictive of reduced overall survival (Table 2). To further demonstrate this, survival curves for patients with or without residual enhancing tumour on MRI adjusted for age, sex, tumour size, insertion of carmustine wafers, and adjuvant therapy, were constructed and revealed significantly better survival for patients without residual tumour (median 13.5 vs. 10.5 months; HR 0.73 [0.56–0.95], p = 0.022; Fig. 2).

Importantly, though the presence of residual 5-ALA fluorescence and residual contrast enhancement on early MRI were consistent in 154 (59.3%) of patients, in a substantial proportion 106 (40.7%) the intraoperative and post-operative assessment of residual disease differed (Table 3).

**Table 3:** Comparison of residual fluorescence with residual enhancement on MRI

	%	Residual MRI enhancement	
		Negative	Positive
Residual 5-ALA fluorescence	Negative	41.0	23.1
	Positive	17.6	18.3

### MGMT and IDH-1 status

Routine clinical evaluation of MGMT methylation status and IDH-1/2 mutation were available for 226 (111 methylated) and 128 (10 mutated) patients, respectively. Surprisingly, MGMT methylation was not significantly associated with survival on univariate analysis (HR 1.06 [0.81–1.39]) and in the multivariable Cox model (Table 2).

For the ten patients with the IDH-1/2 mutation median survival was markedly longer than for the wildtype patients (median 24.1 vs. 12.6 months, log-rank test p = 0.067, likelihood ratio test p = 0.045). The borderline statistical significance presumably reflects the low number of patients with the mutation in this study.

## Discussion

This study has shown that implantation of carmustine wafers does not have an independent effect on survival in patients undergoing 5-ALA guided resection of glioblastoma. In contrast, extent of resection, as evidenced by residual 5-ALA fluorescence and/or residual contrast enhancement on MRI, was found to be an independent prognostic factor for overall survival.

Although the raw unadjusted analyses pointed to a potential benefit of carmustine wafers, when preoperative factors including age, size of tumour, and residual fluorescence, were appropriately controlled for there was no demonstrable effect on outcome. Additional adjustment for post-operative factors, including radiological residual tumour and adjuvant therapy, also did not reveal any benefit of carmustine wafers. Accordingly, the fully adjusted survival curves for patients who did and did not receive carmustine wafers were similar and, within the limits of this study, there did not appear to be a marginal effect of carmustine wafers that might emerge as statistically or clinically significant in a larger cohort. Moreover, carmustine wafers were associated with a tendency to a higher wound complications and infection.

While the implantation of carmustine wafers is an appealing approach to deliver high local concentrations of chemotherapy; they have been shown to provide only limited penetration and degrade over the course of 6–8 weeks [16]. With this pharmacokinetic profile, it may be the case that local carmustine are effective at the resection margins but have no impact on malignant cells penetrating beyond the enhancing disease evident on MRI.

The phase III randomised trial by Stummer and colleagues showed that 5-ALA guided resection nearly doubled the proportion of patients in whom a complete resection was achieved (65 vs. 36%) and this in turn was associated with a significant increase in progression-free survival at 6 months (41.0 vs. 21.1%) [8]. However, the study was designed and conducted prior to temozolomide emerging as the optimal post-surgical treatment of WHO grade IV glioma, and patients allocated to both 5-ALA and control groups received radiotherapy only as first line adjuvant therapy, with chemotherapy reserved until tumour recurrence. In the phase III trial of radiotherapy compared with combined radiotherapy and concomitant and adjuvant temozolomide by Stupp et al. there was a significant survival benefit in the chemotherapy arm (median 14.6 vs. 12.1 months) [17]. Moreover, a post-hoc analysis of the trial data suggested that the greatest benefit of the radiotherapy plus temozolomide regimen was in patients with greater tumour resection [8]. Together, these trials indicate that improved resection directly extends survival and enhances the treatment effect of radical chemo-radiotherapy. Both 5-ALA guided surgery and temozolomide based chemo-radiotherapy are being used increasingly in modern practice with a demonstrated increase in overall survival [18].

In contrast to the widespread adoption of fluorescence-guided surgery and temozolomide-based adjuvant therapy, the role of local chemotherapy in the form of implantable carmustine wafers has remained controversial. Two randomised controlled trials have assessed the efficacy of carmustine wafers inserted at primary surgery for suspected malignant glioma [10]. In the initial small study by Valtonnen et al. randomising 32 patients (27 confirmed as grade IV glioma) a significant survival benefit associated with carmustine wafers was observed. The subsequent phase III study by Westphal et al. enrolled 240 patients and showed an overall 2-month improvement in median survival but the difference between treatment groups for the subset of patients with grade IV glioma was not significant [11]. Moreover, in both trials post-operative treatment was again limited to radiotherapy alone until tumour recurrence. The inconclusive trial findings and the proven efficacy of combined radiotherapy and temozolomide has limited the routine adoption of carmustine wafers as a primary therapy. Nonetheless, a number of reports have detailed the outcomes of multimodal treatment with carmustine wafers followed by combined radiotherapy and temozolomide, and in general suggest longer median overall survival than observed in either the Stupp et al. or Westphal et al. RCTs [19]. However, such observational studies are inherently subject to selection bias. Further, since the introduction of technologies such as 5-ALA to maximise tumour resection, the added benefit of carmustine wafers has become increasingly unclear.

In our centre, 5-ALA guided tumour resection and radical chemoradiotherapy with temozolomide have been the standard of care since 2009. The proportion of patients in whom a complete resection was achieved under 5-ALA guidance was similar to that in the original RCT by Stummer et al. [8]. The overall outcomes in the present cohort are improved over historical data from our unit prior to the introduction of 5-ALA guided resection and temozolomide chemotherapy [20]. Currently we utilise 5-ALA routinely in patients where there is intent to resect tumour to its enhancing margin, if possible around the entire tumour or restricted to regions bordering non-eloquent brain. Intraoperatively, the resection of fluorescent tumour is judiciously assessed against the risk of neurological deficit, incorporating neurophysiological cortical and subcortical mapping and awake testing where appropriate [9]. Higher rates of complete resection with 5-ALA have been reported in other studies but these series are typically highly selected subgroups of patients in whom total resection is deemed possible at the outset [21–23]. No previous studies have examined the effect of carmustine wafers in a treatment programme consisting of 5-ALA guided resection followed by a temozolomide-based chemoradiation: the present findings suggest there is no clinically or statistically significant benefit. In the period since the cohort in this study were treated we have not used carmustine wafers at primary resection of grade IV glioma.

Post-hoc analyses of several trial and data from many retrospective studies support the crucial value of surgical resection and debate the optimal volume of tumour to be removed [24]. Three Cochrane Reviews together with systematic reviews by the European Society for Medical Oncology (ESMO) and the European Association of Neuro-Oncology (EANO) highlight the importance of surgery [2, 3, 25–27]. More recently, compelling prospective data has begun to emerge showing that complete resection confers a survival advantage, although formal prospective data is lacking. A meta-analysis of three randomized phase III trials that recruited a total of 1056 patients concluded, “complete resection appears to improve survival and may increase the efficacy of adjunct/adjuvant therapies” [28]. A prospective clinical trial of 60 grade IV glioma patients showed lack of efficacy of enzastaurin (a PKC and PI-3 kinase/Akt inhibitor) but a secondary analysis revealed a strong prognostic influence of resection on overall survival [29]. A prospective surgical cohort study of 143 grade IV glioma patients showed that the best clinical outcomes were described in those who had complete resection of enhancing disease (median survival for those without residual disease exceeded 24 months, was 16.9 months for those with residual tumour diameter > 0 to ≤ 1 and 13.9 months for those with > 1.5 cm residual disease) and concluded that completeness of resection acts synergistically with radiochemotherapy [4]. A prospective multicenter study of 345 grade IV glioma patients identified GTR as a prognostic factor in multivariate analysis (HR = 0,60; P = 0.003) for overall survival with those undergoing incomplete resection doing no better than those receiving biopsy only [5]. Finally, a retrospective review of 692 patients in the AVAglio trial reported “post-surgical residual enhancing tumor volume is prognostic for OS in newly diagnosed grade IV glioma patients” [30]. Residual tumour identified by intraoperative 5-ALA fluorescence has also been shown to be an independent prognostic indicator in patients with no remaining enhancing disease on MRI [21]. Findings in the present study are consistent with these previous reports: both residual fluorescence at completion of surgery and residual enhancing tumour on MRI were independently associated with reduced survival. When adjusted for other factors, evidence of complete resection on early post-operative MRI extended median survival by approximately 3 months.

The impact of residual enhancing tumour on outcome raises the question of whether early reoperation to complete the resection would be beneficial in selected patients. More research is needed to characterise the volume and patterns of residual tumour on MRI to assess the safety of such an approach i.e. differentiating ‘missed’ and resectable tumour from disease infiltrating eloquent brain with high likelihood of causing neurological deficit. Resecting small volumes of residual tumour may be challenging in the early post-operative setting: anatomical landmarks will be distorted and neuronavigation systems may be less accurate due to brain shift. In addition, the lack of concordance between residual 5-ALA fluorescence and residual on MRI in a minority of patients requires further investigation to determine if there is genuinely enhancing tumour that does not take up 5-ALA or fluorescent disease that does not enhance. A multimodal approach using a combination of technologies to guide resection, including 5-ALA and others such as intraoperative MRI, may offer the best prospect of achieving complete tumour resection [22].

The principal limitation of the present study is its retrospective nature. The propensity score matching technique was employed to mitigate, as best as possible, selection bias and adjust for imbalance in baseline and pre-implantation variables. This procedure extracts matched subgroups of patients and provides the best available approximation to the balanced groups that would be expected in a hypothetical randomised controlled trial allocating patients to receive 5-ALA guided resection with or without the addition of carmustine wafers. The study represents a real-life cohort of neuro-oncology patients; patient selection was not limited to those who received chemo-radiotherapy, reflecting that some patients may not be fit enough for further treatment after surgery. In addition, there was incomplete data for molecular characterisation of all patients in the study and the lack of association between MGMT methylation and survival is difficult to interpret. Variability within and between assays, and controversy around promotor methylation and protein expression are well recognised [31]. Several studies have reported a lack of correlation between MGMT promotor methylation status and clinical outcome [32–36] including a clinical trial [34] and a prospective cohort study [35].
